# Low Iodine Nutrition Knowledge in Chinese Breastfeeding Women despite Adequate Iodine Status

**DOI:** 10.3390/nu16040491

**Published:** 2024-02-08

**Authors:** Shuchang Liu, Andrew Sharp, Steven Lane, Elmer V. Villanueva, Zhiliang Lu, Zheng Feei Ma

**Affiliations:** 1 Department of Biological Sciences, Xi’an Jiaotong-Liverpool University, Suzhou 215123, China; 2 Harris-Wellbeing Research Centre, Faculty of Health & Life Sciences, University of Liverpool, Liverpool L8 7SS, UK; 3 School of Health and Sport Sciences, Liverpool Hope University, Liverpool L16 9JD, UK; 4 Health Data Science, University of Liverpool, Liverpool L69 3BX, UK; 5 School of Science, Xi’an Jiaotong-Liverpool University, Suzhou 215123, China; 6 First Year College, Victoria University, Footscray, VIC 3011, Australia; 7 Centre for Public Health and Wellbeing, School of Health and Social Wellbeing, College of Health, University of the West of England, Bristol BS16 1QY, UK

**Keywords:** iodine, iodine knowledge, iodine status, pregnancy, lactation

## Abstract

There has been a scarcity of evidence about iodine nutrition knowledge among women during pregnancy and lactation. The aim of this study was to determine women’s iodine knowledge and the relationship between knowledge and iodine status during pregnancy and lactation. Women were recruited from a hospital in the western part of China in the third trimester of pregnancy and followed until the end of the first week of lactation. The women’s iodine status was measured by their urinary iodine concentration (UIC) and an iodine-specific, validated food frequency questionnaire (FFQ). Iodine nutrition knowledge was assessed using an iodine nutrition knowledge questionnaire. A total of 200 women (mean age of 29.0 ± 4.2 years) completed the whole study. The majority of the women did not consume enough iodine during both pregnancy and lactation (231.89 vs. 237.26 µg/day). The overall mean iodine knowledge scores in our sample of women during pregnancy and lactation were 4.77 and 4.87, indicating low iodine knowledge. The use of iodized salt and a higher education level were significantly associated with an increased iodine knowledge score. In conclusion, this study reported poor iodine nutrition knowledge in women, highlighting a public health concern. Therefore, the iodine knowledge of women should be improved, possibly via maternal health campaigns to avoid the consequences of iodine deficiency disorders in newborns.

## 1. Introduction

Iodine deficiency is one of the main global public health problems [1]. Adequate dietary iodine intake is needed to ensure the normal production of thyroid hormones, which are crucial for the normal neurodevelopment and growth of the fetus [2]. The general recommendation for iodine intake in normal adults is around 150 µg/day. In comparison, the recommended dietary iodine intake for women during pregnancy and lactation is 250 µg/day, which meets both the iodine requirements of the mother and fetus [3]. This higher iodine intake value reflects a greater demand for iodine in order to maintain healthy thyroid function. Unawareness of this fact could lead to a higher risk of undereating during pregnancy and lactation. Therefore, inadequate dietary iodine intake during pregnancy and lactation may cause adverse health consequences and lead to the development of iodine deficiency disorders (IDDs) [4].

Since China introduced the universal salt iodization (USI) programme in 1994, iodine status in the Chinese population improved significantly [5]. According to a nationally representative cross-sectional study covering 31 regions of mainland China after 20 years of USI, the coverage of iodized salt was found to be 95.37% [6]. Goitre prevalence was 1.17% down from 20.40% and the median urine iodine concentration (UIC) was 177.89 μg/L in adult populations [6,7]. At the same time, there was a gradual increase in IDD awareness among the Chinese population, largely driven by the implementation of USI, as well as health promotion and education initiatives [8,9]. Despite these efforts, there has been no improvement in knowledge about iodine nutrition [10,11,12]. Additionally, the iodine status of pregnant and lactating women has not been addressed [6]. Having adequate knowledge about iodine nutrition may help women proactively consume iodine from iodine-rich diets during pregnancy and lactation, meeting the iodine requirements of both the mother and fetus [10]. Additionally, increasing public awareness and understanding of iodine nutrition contributes to a reduction in IDDs in populations [13]. Since iodine can only be obtained from diets, information about iodine requirements during pregnancy and lactation may be important to prevent suboptimal iodine intake and improve women’s knowledge of iodine [10,11]. In China, this information is not often communicated to pregnant and lactating women because it is generally assumed that women can obtain enough iodine from their diet because of the mandatory universal salt iodization. A lack of iodine nutrition knowledge may be one of the risk factors for iodine deficiency in pregnant and lactating women [14,15,16,17]. In addition, low iodine knowledge has been associated with a poorer iodine status in pregnant and lactating women [18,19].

Shaanxi is located in the Western part of China; it is far from the sea and was previously identified as one of the endemic areas for IDDs before the implementation of USI [20,21]. The sex-adjusted incidence rate of congenital hypothyroidism in Shaanxi was 3.9 per 10,000 newborns screened [22], which was higher than in some developed countries including New Zealand (3.1 per 10,000 newborns screened) and France (2.9 per 10,000 newborns screened). The prevalence of iodine deficiency in pregnant Chinese women was reported to be 48.2% [23]. In China, pregnant women are provided with nutritional education or even supplements by health professionals, without a specific emphasis on iodine. For example, maternal and child health hospitals provide free folic acid supplements to women during the three months before pregnancy and the first three months of pregnancy [24]. In addition, due to the differences in dietary habits, economic conditions and soil iodine concentrations in different areas of China, knowledge about iodine nutrition in this population needs to be improved.

In China, limited research has been conducted on iodine knowledge during pregnancy and lactation. There is a notable gap in the literature as no studies have yet investigated whether women’s iodine knowledge changes over time, from pregnancy to lactation. Therefore, the aim of this study was to determine knowledge about iodine nutrition and the predictors of iodine nutrition knowledge scores, as well as their relationship with the iodine status of women during pregnancy and lactation.

## 2. Materials and Methods

This study was conducted between May 2021 and May 2022 in Xianyang Central Hospital Affiliated with the Medical Department of Xi’an Jiaotong University, Shaanxi, China. Pregnant women were considered eligible for the inclusion in this study if they were of Chinese nationality, in their third trimester of pregnancy, aged between 18–50 years, had lived in Shaanxi for at least one year, and could read and write in Chinese. Pregnant women were excluded from the study if they had a history of thyroid disease. Participants were followed up until their completion of the first week of the lactation period.

All participants gave informed consent before they participated in the study. This study was conducted following the ethical standards of the Xi’an Jiaotong-Liverpool University Ethics Committee (reference no. 20-01-09). The results of this study were reported following the guidelines of the STrengthening the Reporting of OBservational studies in Epidemiology (STROBE) Checklist for cohort studies [25,26].

### 2.1. Iodine Nutrition Knowledge Questionnaire

Participants were required to complete a socio-demographic questionnaire including their age and history of thyroid disease. In addition, participants were asked about their understanding of iodine nutrition with the following questions: “Do you know what iodine is?” and “I think I get enough iodine through my diet”. Participants also completed a questionnaire assessing their iodine knowledge [11,19,27]. Questions about iodine knowledge included the following: “Which part of the body needs iodine to produce hormones?”, “Which of the following foods are the most important dietary iodine sources? (multiple choice)”, “Iodine is important for? (multiple choice)”, “What is the maternal iodine status in China? (multiple choice)”, and “In China, which population groups are routinely recommended to take an iodine supplement? (multiple choice)”. Participants were asked to provide a response for whether they had received iodine education previously. The questionnaire used was adapted from previous published studies [11,19,27] and reviewed by an expert in iodine status assessment. The questionnaire was subsequently validated in the study setting and pilot-tested to further check for content comprehension, validity, and clarity. To ensure the quality of the translation, back-translation was performed and reviewed by hospital staff in the Xianyang Central Hospital Affiliated with the Medical Department of Xi’an Jiaotong University Ethics Committee. Pre-testing was also conducted at the hospital site to assess the quality of the translations in terms of readability, comprehensibility, and relevance to evaluate the face validity. Participants were required to complete the questionnaire twice, both during their third trimester of pregnancy and the first week of lactation.

The iodine nutrition knowledge variables were calculated as the total iodine nutrition knowledge scores for participants (range 0 to 12). The iodine nutrition knowledge scores were divided into five different categories: no nutrition knowledge (0 point), poor nutrition iodine knowledge (0–3 points), low iodine nutrition knowledge (4–6 points), medium iodine nutrition knowledge (7–9 points), and high iodine nutrition knowledge (10–12 points) [19].

### 2.2. Estimated Daily Iodine Intake

Participants were asked to fill in a questionnaire containing questions on socio-demographics, namely a 33-item iodine-specific semi-quantitative FFQ. The FFQ consisted of foods that are the major dietary food sources of iodine consumed by the Chinese population, including buns, noodles, porridge, eggs, bean products, soybean milk products, fish, and seafoods [28]. In addition, questions on the use of iodized salt were included. Participants were required to choose the appropriate choice according to the frequency and serving size of the foods consumed over the previous week [28]. The frequency choices for each of the food items were as follows: “never”, “less than once a month”, 1–3 times per month”, “once a week”, “2–4 times per week”, “5–6 times per week”, “once per day”, “2–3 times a day”, “4–5 times a day”, and “6 or more times a day” [28]. The iodine intake from each food item was calculated using the 2019 Chinese Food Composition Tables [29].

### 2.3. Maternal Adherence with Recommendations for Iodine Supplements

In addition, the participants were asked to complete 13 questions on the adherence of women to the recommendations regarding iodine supplements twice, during their 3rd trimester of pregnancy and 1st week of postpartum. The questions included the following: “Do you take supplements?”, “Have you ever been prescribed a maternal supplement by a health professional?”, “How often do you/did you take a maternal supplement?”, “Thinking about breastfeeding, what did you do/plan to do (do you plan to take maternal supplements during the postpartum period?)”, and “How important do you consider these nutrients (folic acid, iron, iodine, vitamin D) from a maternal supplement for pregnancy and postpartum periods?”.

The questionnaire should have taken approximately 15–20 min to complete. The iodine-specific FFQ and questions on maternal adherence with recommendations for iodine supplements were pilot-tested, back-translated from English to Chinese, and validated in a group of Chinese pregnant women for content validity, comprehension, and clarity [28].

### 2.4. Assessment of Urinary Iodine Concentration (UIC)

Participants were required to provide one spot morning urine sample during the third trimester of pregnancy and two spot morning urine samples during the first week of lactation. All urine samples from the same participants were analyzed in the same batch for their UIC based on the Sandell–Kolthoff reaction, as described previously [30].

### 2.5. Statistical Analysis

The study data were analyzed using SPSS statistical software package version 25.0 (IBM Corp., Armonk, NY, USA). The mean and SD values were used to report the normally distributed variables, while the median (25th, and 75th percentiles) was used to describe the non-normally distributed variables. McNemar’s test was used to test for between-group differences for nominal paired data. For related samples, the paired *t*-test and Wilcoxon rank test were used to compare the means of normally distributed variables and the medians of non-normally distributed variables, respectively. Binary logistic regression analyses were employed to explore the predictors of maternal iodine knowledge and intake. Linear regression was also used to examine the dietary iodine intake of women during pregnancy and lactation as dependent variables, with the consumption of the food groups used as independent variables. A two-sided *p*-value of <0.05 was used to indicate statistical significance.

The total dietary iodine intake from each of the 33 major food groups was calculated for each participant. The participants who reported that they used iodized salt in their diet had 150 μg of iodine added to their total dietary iodine intake.

## 3. Results

A total of 200 women participated in this longitudinal cohort study and were assessed in their third trimester of pregnancy and again in their first week of lactation. Of those women, the overall median UIC of women in our sample during pregnancy was 112 μg/L (85, 134 μg/L), indicating the presence of maternal iodine deficiency (median UIC cut-off of <150 μg/L). On the other hand, the median UIC of women in our sample during lactation was 113 μg/L (90, 133 μg/L), suggesting maternal iodine sufficiency (median UIC cut-off of ≥100 μg/L). The overall mean dietary iodine intakes of women in our sample for pregnancy and lactation calculated from the FFQ were 231.89 ± 146.02 and 237.26 ± 156.20 µg/day, respectively. The overall mean iodine intakes during both pregnancy and lactation were categorized as iodine deficient (i.e., iodine intake ≤250 µg/day) [31]. Although both pregnant and lactating women have the same recommended iodine intake of 250 μg/day, lactating women have a lower median UIC cut-off of ≥100 μg/L than pregnant women (≥150 μg/L) for determining iodine sufficiency. This is because the lower median UIC cut-off in lactating women accounts for iodine excretion in breast milk [32]. During pregnancy, 70.0% of women did not achieve the recommended iodine intake of 250 µg/day, and this figure remained similar until lactation (69.0% of women had an iodine intake ≤250 µg/day). Overall, 65.0% of women reported the use of iodized salt in their diet during pregnancy and lactation. Table 1 shows the sociodemographic characteristics of women in our sample.

For both pregnancy and lactation, the aquatic products group contributed the highest percentage to the daily iodine intake of the women, with 123.47 µg (91.36%) in pregnancy and 131.10 µg (92.16%) in lactation. Other food groups including beans and bean products, meat, eggs, nuts, staple food, milk and dairy products, fruits, and snacks contributed a relatively low percentage to the daily iodine intake of the women during both pregnancy and lactation. The consumption of eggs contributed significantly more to daily iodine intake in pregnancy compared to lactation (0.60% vs. 0.53%) (*p* = 0.039). However, no differences were reported between pregnancy and lactation in the contribution of other food groups to the women’s daily iodine intake.

### 3.1. Use of Maternal Supplements

Overall, during pregnancy and lactation, 33.5% of women reported using supplements. Of those using any type of dietary supplements, 30% reported using non-iodine-containing dietary supplements, 3.5% reported using only iodine-containing dietary supplements, and 66.5% reported not using any type of dietary supplements. For women using maternal supplements, 22% reported being prescribed a maternal supplement by a health professional, while 78% self-purchased without a prescription. More than one-third of women (35.5%) reported using maternal dietary supplements during pregnancy, 5.5% reported taking supplements that were not maternal supplements, 2.5% reported using maternal supplements before pregnancy, 2% reported using a herbal remedy specifically for pregnancy or breastfeeding, and 54% reported not taking any supplements. Regarding the frequency of taking a maternal supplement, only 40% of women answered this question. Among those who did respond, the most common response was “every day” (15.5%), followed by “3–4 times a week” (10.5%), “almost every day” (5%), “1–2 times a week” (4%), and “less than once a week” (4%).

In total, 55% of the women reported not knowing what they planned to do regarding taking maternal supplements while breastfeeding. Among those who did respond, the most common response was planning not to take maternal supplements while breastfeeding (28.5%), followed by planning to take maternal supplements for a longer time than breastfeeding (11.5%), and planning to take maternal supplements for less time than breastfeeding (5%).

### 3.2. Maternal Iodine Knowledge

Regarding the ‘folic acid for pregnancy’ option, almost all women (98.5% during pregnancy and 98.5% during lactation) perceived folic acid as important (Table 2). Similarly, a high percentage of women (88.5% during pregnancy and 89.0% during lactation) perceived iron as important during pregnancy. Most women (75.0% during pregnancy and 77.0% during lactation) perceived vitamin D as important during pregnancy. For the ‘iodine for pregnancy’, a significantly lower percentage of women during pregnancy (67.5%) compared to lactation (70.5%) perceived it as important (*p* = 0.031).

More than half of the women (65.5% during pregnancy and 68.5% during lactation) perceived folic acid as important during breastfeeding. The majority of the women (75.5% during pregnancy and 76.0% during lactation) perceived iron as important during breastfeeding. Most women perceived iodine (65.0% during pregnancy and 67.0% during lactation) and vitamin D (69.5% during pregnancy and 72.0% during lactation) as important during breastfeeding.

There were 36.5% of women who reported that they received iodine education throughout pregnancy and lactation. About 65% of women reported that they used iodized salt at home and this proportion remained similar throughout pregnancy and lactation (Table 3). More than half of women (68.5% during pregnancy and 70.0% during lactation) reported that they knew what iodine is. When asked if they thought they received enough iodine, most women (59.5% during pregnancy and 60.5 during lactation) agreed that they received enough iodine. There was no difference in the responses ‘do you know what iodine is’ and ‘I think I get enough iodine’ between women in pregnancy and lactation.

More than half of women (58.0% during pregnancy and 59.5% during lactation) correctly identified the thyroid gland as the part of the body that needs iodine to produce thyroid hormones; meanwhile, about a third of women during pregnancy (38.0%) and lactation (36.5%) did not know which part of the body needed iodine to produce thyroid hormones (Table 4). The majority of women identified iodized salt (both 80.5% during pregnancy and lactation), followed by fish and seafood (60.0% during pregnancy and 60.5% during lactation), milk (13.5% during pregnancy and 14.0% during lactation), nuts (13.0% during pregnancy and 12.5% during lactation), meat (both 10.5% during pregnancy and lactation), fruit (10.5% during pregnancy and 10.0% during lactation), vegetable oil (6.5% during pregnancy and 6.0% during lactation) and bread (2.5% during pregnancy and 2.0% during lactation) as important dietary sources of iodine; meanwhile, about 10% of women during pregnancy (11.5%) and lactation (11.0%) did not know which sources are considered important dietary sources of iodine.

The majority of women (43.5% during pregnancy and 45.5% during lactation) correctly identified that ‘iodine is important for normal child growth and development’; 33.0% during pregnancy and 34.0% during lactation correctly identified that ‘iodine is important for maintaining normal metabolism’; 27.0% during pregnancy and 28.0% during lactation correctly identified that ‘iodine is important for preventing mental health retardation’; and 24.0% during pregnancy and 24.0% during lactation correctly identified tat ‘iodine is important for normal fetal development’. The option ‘don’t know if iodine is important for’ was selected by about one-third of women (40.0% during pregnancy and 38.0% during lactation).

More than half of the women (56.0% during pregnancy and 56.5% during lactation) thought that the maternal iodine status in China was ‘too low intake, which was a problem earlier, but not now’, followed by a ‘too low intake is a current problem’ (27.5% during pregnancy and 26.5% during lactation) and a ‘too high intake is a problem now’ (16.5% during pregnancy and 17.0% during lactation). Regarding the question about which target population groups are regularly suggested to take an iodine supplement, nearly half of women chose ‘pregnant women’, while a third of women chose ‘breastfeeding women’ and ‘all women of childbearing age’; only a smaller percentage of women (around 15.0%) chose ‘all babies’.

The mean scores of iodine knowledge in women during pregnancy were significantly lower than during lactation (4.77 ± 2.95 vs. 4.87 ± 2.96) (*p* = 0.004) (Table 5). The ‘none-to-medium’ category represents the percentage of participants who scored in the lower half of the range of possible scores. In this study, 90.0% of the participants scored in this category. The majority of women in our sample were reported to have none to low iodine knowledge scores (73.0% and 71.0% during pregnancy and lactation, respectively), followed by 17.0% and 19.0% during pregnancy and lactation, respectively, with medium scores. Only a small percentage of women during pregnancy (10.0%) and lactation (10.0%) scored in the ‘high’ category.

Table 6 presents the predictors of the iodine nutrition knowledge scores of women during pregnancy and lactation. The significant predictors are the use of iodized salt, education and dietary iodine intake among pregnant women, and the use of iodized salt and dietary iodine intake among lactating women. This suggested that the pregnant and lactating women who did not use iodized salt were half as likely to have higher iodine knowledge scores when compared to women who use iodized salt. Similarly, if a pregnant woman had a higher education level, the woman was twice as likely to have a greater knowledge of iodine. However, if a lactating woman had a higher iodine intake, the woman was twice as likely to have better iodine knowledge; meanwhile, a pregnant woman was 0.3 times more likely to have better iodine knowledge. After adjustment for variables of age, UIC pregnancy, the use of iodized salt, occupation, education, BMI and iodine intake in the model, the predictors were unchanged among pregnant women; only the use of iodized salt and education level were significant predictors of the iodine nutrition knowledge scores of lactating women.

## 4. Discussion

This study assessed the iodine nutrition knowledge of women during pregnancy and lactation. The findings of this study indicated that the majority of women in our sample had none-to-low knowledge of iodine nutrition during pregnancy and lactation, with mild iodine deficiency during pregnancy. Poor iodine nutrition knowledge in women during these critical periods has also been documented in other countries, including Australia, Norway and New Zealand [18,19,33,34]. While the adverse health effects of severe maternal iodine deficiency are well-known, the impact of mild maternal iodine deficiency is often overlooked. However, studies have reported that mild iodine deficiency during pregnancy may also lead to the development of adverse health effects, including impaired cognitive skills in infants [34,35,36,37]. Therefore, the adverse health effects of mild maternal iodine deficiency are no less important.

Evidence of inadequate iodine nutrition knowledge and iodine status in pregnancy and lactation has been reported in several developed countries including the UK, Norway, Australia, and New Zealand [16,18,19,38,39]. This may reflect the low public health priority of iodine nutrition within these countries. For example, in Australia, although iodized salt has been available for decades, the consumption of iodized salt has not been broadly accepted [18,34,38], possibly due to a lack of public education about the benefits of using iodized salt [40]. In addition, advice on the use of iodine supplements during pregnancy and lactation at antenatal services in China appears to be lacking [24]. This factor, in combination with the cost of iodine supplements, may lead to the inadequate implementation of the iodine supplementation guidelines. Therefore, the confusion around the need for taking iodine supplements during pregnancy and lactation, with the lack of public health initiatives aimed at reducing the consequences of iodine deficiency, may translate into an inadequate iodine status in women throughout pregnancy and lactation. Women may then enter pregnancy and lactation in an iodine-deplete state. They may not be able to make informed dietary choices and supplementation practices to improve their iodine status during pregnancy and lactation.

Studies investigating knowledge about iodine nutrition knowledge and its relationship with iodine status in pregnant and lactation are limited in China [10,11,41,42]. Of these four studies, three studies only focused on pregnant women [11,41,42], and one study included both pregnant and lactating women [10]. A study by Wang et al. described that, in Zhejiang province, the mean iodine nutrition knowledge scores of iodine-sufficient pregnant women in coastal regions were lower than those of iodine-deficient pregnant women in inland regions (24.2 points vs. 25.0 points) (*p* < 0.001) [11]. However, there was no cut-off used to indicate poor iodine knowledge in the study by Wang et al. [11]. In Shanghai, of 354 iodine-deficient pregnant women, low iodine nutrition knowledge scores were reported in 52.4% (76/145), 42.6% (40/94) and 45.0% (49/109) of women in the first, second and third trimesters of pregnancy [42]. A cut-off point of <6 was used to indicate low iodine nutrition knowledge scores, and the majority of women in the first trimester of pregnancy had lower iodine nutrition knowledge scores than women in the second and third trimesters of pregnancy [42]. Another study in Shanghai reported that 30.3% of the iodine-deficient pregnant women lacked iodine nutrition-related knowledge, and a cut-off point of <5 was used to denote poor iodine nutrition knowledge scores [41]. A study conducted in Xinjiang, China, by Nie et al. reported that pregnant women had a significantly higher mean iodine knowledge score than lactating women (12.59 vs. 12.57) (*p* < 0.05) [10]. Although both pregnant and lactating women were iodine-sufficient, most women (73.4% of pregnant women and 82.5% of lactating women) in Xinjiang were reported to have a medium level of iodine nutrition knowledge [10]. Due to the different measurement scales and cut-off points used in assessing iodine nutrition knowledge, a direct comparison of these results is impossible.

In this study, women during pregnancy had a significantly lower mean score of iodine nutrition knowledge than during lactation, which is similar to the results reported by Garnweidner-Holme et al. in Norway (7 vs. 12) (*p* < 0.001) [19]. Although it is acknowledged that there was a small difference between both iodine nutrition scores for women during pregnancy and lactation, the improved knowledge of the importance of iodine from pregnancy to lactation could be a bias from the study design. This is because this study implied that iodine was important when approaching these women. The policies implemented to tackle IDDs in the Chinese population via USI changed before and after the successful elimination of IDDs in 2011. During the period of tackling IDDs (before 2011), healthcare workers actively provided iodine nutrition information and the USI programme, which contributed to an increase in the use of iodized salt to ≥95%. After successfully eliminating IDDs in 2011, IDD surveillance reported the presence of more-than-adequate iodine nutrition in the Chinese population and an increased incidence of thyroid diseases in coastal provinces [43], which was due to the high consumption of iodized salt [44,45,46,47,48]. However, the fact that the high incidence of thyroid disease was due to the consumption of iodized salt may be exaggerated and such findings were based on the data from hospital records [11]. Since then, concerns regarding IDDs have shifted to the prevention of excessive iodine nutrition in the population. Both the vigilance and financial fund for eliminating IDDs provided by the government were dropped sharply [11]. Therefore, all these factors may have contributed to poor iodine nutrition knowledge in the Chinese population. Especially for pregnant women, public opinion may make them cautious about excessive iodine intake, but this overlooks their special requirement of nearly double the amount of iodine compared to the general adult population.

The present study showed that 33.5% of women reported using supplements for pregnancy and lactation; of these, only 3.5% took an iodine-containing supplement, which was higher than 0.92% in Shanghai, but lower than other countries [49,50,51,52,53]. In the USA, 72.2% of pregnant women reported that they consumed a dietary supplement; of these, 17.8% of them took an iodine-containing supplement [50]. In Switzerland, the proportion of pregnant women taking iodine-containing supplements ranged from 9% to 15% [51,52,53]. For lactating women in the USA, 75.0% used a dietary supplement; of these, 19.0% consumed an iodine-containing supplement [50].

In this study, the use of iodized salt, education level, and iodine intake were found to predict iodine nutrition knowledge scores during pregnancy. Similarly, the use of iodized salt and education level were shown to predict iodine nutrition knowledge scores during lactation. Despite 80.5% of women correctly identifying iodized salt as one of the main iodine sources, only 64.5% used iodized salt at home, which was lower than the 95.9% reported in China’s IDD survey in 2019, but similar to the consumption rate of iodized salt (71.5%) in Shanghai [41]. Our study suggests that there may be a discrepancy between iodine nutrition knowledge and the actual iodine intake among women. Also, the lack of knowledge about iodine nutrition knowledge may have been somewhat offset by other maternal nutrition education topics including folate and sodium (salt), which may have resulted in the low consumption of iodized salt. However, it should be noted that given the low consumption rate of iodized salt, iodine nutrition education is important for women to sustain their iodine status at a sufficient level throughout pregnancy and lactation. Although universal salt iodization has been introduced by the Chinese regulatory authorities, it is possible for the women to purchase non-iodized salt from the market due to the relaxed availability of non-iodized salt. Therefore, some women might have chosen to use non-iodized salt because of the concerns about an excessive iodine intake affecting the development of the fetus. In this study, the iodine nutrition knowledge of almost all women did not change throughout pregnancy and lactation. Therefore, it is possible that some women were influenced by incorrect iodine nutrition knowledge and unaware of this, leading to a poor iodine status throughout pregnancy and lactation. Health approaches to improving the maternal iodine status of women should include improving their iodine nutrition knowledge and dietary iodine intake.

This study has several strengths. This study adapted and piloted the iodine nutrition knowledge questionnaire from Garnweidner-Holme et al. [19], which allowed comparisons of results between studies that employed similar methodological approaches in assessing iodine nutrition knowledge. The majority of women in this study had a university-level education. Considering the insufficient iodine status and nutrition knowledge observed among these women during pregnancy, those with lower levels of education may have an even poorer maternal iodine status [35]. Although our study included a cohort of women followed up from pregnancy to lactation, the follow-up period during lactation was only one week. A further limitation was that this study was based on self-reported maternal information, which was obtained from the questionnaires. There might be differences between self-reported and actual behavior, which might lead to misreporting and conservative estimates of iodine nutrition knowledge scores. Future studies should consider the reasons why women during pregnancy and lactation take supplements without medical prescriptions. However, this study has addressed the research gaps by providing insights into the iodine knowledge levels of women during pregnancy and lactation in the Western part of China.

## 5. Conclusions

This study highlighted that iodine nutrition knowledge among Chinese women during pregnancy and lactation is not at the desired level. Considering the low consumption of iodized salt, dietary iodine intake alone is inadequate to cover the increased iodine requirements of both mothers and fetuses. Therefore, it is crucial to enhance iodine nutrition knowledge among women during pregnancy and lactation through means such as maternal nutritional education campaigns.

## Figures and Tables

**Table 1:** Sociodemographic characteristics of participants who were followed from pregnancy to lactation (n = 200).

Characteristics	Value *
Age (years)	29.0 ± 4.2
Gestational age (week)	37.0 ± 2.4
Delivery age (week)	39.9 ± 0.8
Height (cm)	161.0 ± 4.8
Pre-pregnancy weight (kg)	55.9 ± 8.4
Weight gain	14.9 ± 3.7
BMI (kg/m^2^)	21.5 ± 2.8
*Blood pressure* (mmHg)	
Systolic pressure	115.1 ± 10.5
Diastolic pressure	74.9 ± 7.3
*Education level*	
Below university level	69 (34.5)
University level and above	131 (65.5)
*Occupation*	
Employed	164 (82.0)
Non-employed	36 (18.0)
*Smoking status*	
Smoker	3 (1.5)
Non-smoker	197 (98.5)

* Data are means ± SD or n (%). BMI, body mass index.

**Table 2:** Percentages of the perceived importance of nutrients by women.

Variables	Pregnancy, n (%)	Lactation, n (%)	*p*-Value
Folic acid for pregnancy	197 (98.5)	197 (98.5)	>0.999
Iron for pregnancy	177 (88.5)	178 (89.0)	>0.999
Iodine for pregnancy	135 (67.5)	141 (70.5)	**0.031**
Vitamin D for pregnancy	150 (75.0)	154 (77.0)	0.125
Folic acid for breastfeeding	131 (65.5)	137 (68.5)	0.070
Iron for breastfeeding	151 (75.5)	152 (76.0)	>0.999
Iodine for breastfeeding	130 (65.0)	134 (67.0)	0.125
Vitamin D for breastfeeding	139 (69.5)	144 (72.0)	0.063

Bold values denote statistical significance at the *p* < 0.05 level.

**Table 3:** Understanding of iodine during pregnancy and lactation.

Variables	Pregnancy (n = 200), n (%)	Lactation (n = 200), n (%)	*p*-Value
*Received iodine education before*			
Yes	73 (36.5)	73 (36.5)	>0.999
No	127 (63.5)	127 (63.5)	
*Do you think the salt you take at home is iodized?*			
Yes	129 (64.5)	130 (65.0)	>0.999
No	71 (35.5)	70 (35.0)	
*Do you know what iodine is*			
Yes	137 (68.5)	140 (70.0)	0.250
No	63 (31.5)	60 (30.0)	
*I think I get enough iodine*			
Agree	119 (59.5)	121 (60.5)	0.500
Disagree	81 (40.5)	79 (39.5)	

**Table 4:** Knowledge regarding iodine’s dietary sources, functions, and national iodine status of women during pregnancy and lactation.

Iodine Knowledge	Pregnancy (n = 200), n (%)	Lactation (n = 200), n (%)	*p*-Value
*Which part of the body needs iodine to produce thyroid hormones?*			
Brain	14 (7.0)	13 (6.5)	>0.999
Heart	12 (6.0)	11 (5.5)	>0.999
Bone	16 (8.0)	16 (8.0)	>0.999
Thyroid gland	116 (58.0)	119 (59.5)	0.125
Don’t know	76 (38.0)	73 (36.5)	0.500
*Most important dietary iodine sources*			
Meat	21 (10.5)	21 (10.5)	>0.999
Milk	27 (13.5)	28 (14.0)	>0.999
Fruit	21 (10.5)	20 (10.0)	>0.999
Fish and seafood	120 (60.0)	121 (60.5)	>0.999
Bread	5 (2.5)	4 (2.0)	>0.999
Vegetable oil	13 (6.5)	12 (6.0)	>0.999
Nuts	26 (13.0)	25 (12.5)	>0.999
Iodized salt	161 (80.5)	161 (80.5)	>0.999
Don’t know	23 (11.5)	22 (11.0)	>0.999
*Iodine is important for?*			
Normal child growth and development	87 (43.5)	91 (45.5)	0.125
Prevent blindness	13 (6.5)	12 (6.0)	>0.999
Normal fetal development	48 (24.0)	48 (24.0)	>0.999
Strength in teeth and skeleton	43 (21.5)	42 (21.0)	>0.999
Maintain normal metabolism	66 (33.0)	68 (34.0)	0.500
Prevent spina bifida	17 (8.5)	17 (8.5)	>0.999
Prevent mental retardation	54 (27.0)	56 (28.0)	0.250
Don’t know	80 (40.0)	76 (38.0)	0.250
*Maternal iodine status in China*			
Too low intake is a current problem	55 (27.5)	53 (26.5)	0.500
Too high intake is a current problem	33 (16.5)	34 (17.0)	>0.999
Too low intake was a problem earlier, not now	112 (56.0)	113 (56.5)	>0.999
*In China, which population groups are routinely recommended to take an iodine supplement?*			
Pregnant women	92 (46.0)	96 (48.0)	0.125
Breastfeeding women	69 (34.5)	71 (35.5)	0.250
All women of childbearing age	62 (31.0)	61 (30.5)	>0.999
All babies	29 (14.5)	30 (15.0)	>0.999

**Table 5:** Iodine knowledge scores of women during pregnancy and lactation.

Iodine Knowledge Score	Pregnancy (n = 200)	Lactation (n = 200)	*p*-Value
Total score	4.77 ± 2.95	4.87 ± 2.96	**0.004**
*None-to-medium*			0.125
None	11 (5.5)	11 (5.5)	
Poor	60 (30.0)	59 (29.5)	
Low	75 (37.5)	72 (36.0)	
Medium	34 (17.0)	38 (19.0)	
*High*			
High	20 (10.0)	20 (10.0)	

Data are means ± SD or n (%). Bold values denote statistical significance at the *p* < 0.05 level.

**Table 6:** Predictors of iodine knowledge score among pregnant and lactating women.

	Pregnant Women (n = 200)						Lactating Women (n = 200)					
	Unadjusted Coef.			Adjusted Coef. ^k^			Unadjusted Coef.			Adjusted Coef. ^k^		
	β	OR	95% CI	β	OR	95% CI	β	OR	95% CI	β	OR	95% CI
Age ^a^	−0.023	0.977	0.520, 1.837	−0.025	0.976	0.484, 1.967	0.006	1.006	0.543, 1.864	−0.070	0.932	0.476, 1.826
UIC pregnancy ^b^	−0.503	0.605	0.234, 1.567	−0.514	0.598	0.212, 1.689	0.616	0.540	0.209, 1.395	−0.748	0.473	0.173, 1.298
UIC lactation ^c^	-	-	-	-	-	-	0.018	1.018	0.539, 1.921	-	-	-
BMIC ^d^	-	-	-	-	-	-	0.078	1.081	0.420, 2.783	-	-	-
Use of iodized salt ^e^	−0.677	0.508	0.337, 0.766 *	−0.690	0.502	0.328, 0.767 *	−0.746	0.474	0.315, 0.714 *	−0.737	0.479	0.307, 0.745 *
Occupation ^f^	0.214	1.238	0.562, 2.728	0.466	1.593	0.666, 3.815	0.249	1.283	0.593, 2.776	0.395	1.484	0.640, 3.441
Education ^g^	0.807	2.242	1.088, 4.617 *	0.968	2.631	1.202, 5.760 *	0.687	1.987	0.997, 3.960	0.784	2.191	1.027, 4.673 *
BMI ^h^	−0.093	0.911	0.422, 1.968	−0.066	0.936	0.410, 2.138	−0.218	0.805	0.374, 1.730	−0.229	0.796	0.353, 1.792
Use of supplements ^i^	−0.343	0.710	0.374, 1.345	-	-	-	−0.328	0.720	0.384, 1.351	-	-	-
Iodine intake ^j^	−1.157	0.314	0.138, 0.716 *	−1.202	0.301	0.127, 0.710 *	0.658	1.932	1.017, 3.668 *	0.127	1.135	0.551, 2.340

^a^ Categories for age: 0 = age < 30, 1 = age ≥ 30. ^b^ Categories for UIC pregnancy: 0 = UIC pregnancy < 150 µg/L, 1 = UIC pregnancy ≥ 150 µg/L. ^c^ Categories for UIC lactation: 0 = UIC lactation < 100 µg/L, 1 = UIC lactation ≥ 100 µg/L. ^d^ Categories for BMIC: 0 = BMIC = 60–465 µg/L, 1 = BMIC < 60 or >465 µg/L. ^e^ Categories for use of iodized salt: 0 = use, 1 = not use/unknown. ^f^ Categories for Occupation: 0 = employed, 1 = non-employed. ^g^ Categories for education: 0 = completed high school or lower education, 1 = higher education. ^h^ Categories for BMI: 0 = normal weight, 1 = under/overweight. ^i^ Categories for use of supplements: 0 = use, 1 = not use. ^j^ Categories for iodine intake: 0 = inadequate, 1 = adequate. ^k^ Adjusted for variables of age, UIC pregnancy, use of iodized salt, occupation, education, BMI and iodine intake in the model. Dependent variable: iodine knowledge during pregnancy and lactation. * significant (*p* < 0.05). OR, odd ratio; CI, confidence interval; UIC, urinary iodine concentration; BMIC, breast milk iodine concentration, BMI, body mass index; Coef, coefficient. Bold values denote statistical significance at the *p* < 0.05 level.

## Data Availability

Data available upon request from the corresponding authors.

## References

[1] Lazarus J.H. (2015). The importance of iodine in public health. Environ. Geochem. Health.

[2] Skeaff S.A. (2011). Iodine deficiency in pregnancy: The effect on neurodevelopment in the child. Nutrients.

[3] Secretariat W.H.O., Andersson M., De Benoist B., Delange F., Zupan J. (2007). Prevention and control of iodine deficiency in pregnant and lactating women and in children less than 2-years-old: Conclusions and recommendations of the Technical Consultation. Public Health Nutr..

[4] Pearce E.N. (2012). Effects of iodine deficiency in pregnancy. J. Trace Elem. Med. Biol..

[5] Yang L., Li M., Liu X., Wu M., Zhang J., Zhao L., Ding G., Yang X. (2020). Evaluation of iodine nutritional status among pregnant women in China. Thyroid.

[6] Liu T., Li Y., Teng D., Shi X., Yan L., Yang J., Yao Y., Ye Z., Ba J., Chen B. (2021). The characteristics of iodine nutrition status in China after 20 years of USI: An epidemiology study covering 31 provinces. Thyroid Off. J. Am. Thyroid Assoc..

[7] Ma T., Guo J., Wang F. (1993). The epidemiology of iodine-deficiency diseases in China. Am. J. Clin. Nutr..

[8] Albert B.B., Cutfield W.S., Webster D., Carll J., Derraik J.G.B., Jefferies C., Gunn A.J., Hofman P.L. (2012). Etiology of increasing incidence of congenital hypothyroidism in New Zealand from 1993–2010. J. Clin. Endocrinol. Metab..

[9] Barry Y., Bonaldi C., Goulet V., Coutant R., Léger J., Paty A.-C., Delmas D., Cheillan D., Roussey M. (2016). Increased incidence of congenital hypothyroidism in France from 1982 to 2012: A nationwide multicenter analysis. Ann. Epidemiol..

[10] Nie J., Zhu Y., Wang C., Lin Q., Tayier R., Cai Z., Ma P., Zhang L. (2023). Relationship between iodine knowledge and dietary iodine intake in pregnant and lactating women: A cross-sectional study. Public Health Nutr..

[11] Wang X., Lou X., Mo Z., Xing M., Mao G., Zhu W., Wang Y., Chen Y., Wang Z. (2019). Poor iodine knowledge, coastal region, and non-iodized salt consumption linked to low urinary iodine excretion in Zhejiang pregnant women. Nutrients.

[12] Jin Y., Luo X., Ma Z.F., Dong Z., Carciofo R., Li X., Skeaff S. (2020). Adequate iodine intake among young adults in Jiangsu Province, China despite a medium iodine knowledge score. Eur. J. Investig. Health Psychol. Educ..

[13] Hatch-McChesney A., Lieberman H.R. (2022). Iodine and iodine deficiency: A comprehensive review of a re-emerging issue. Nutrients.

[14] Axford S., Charlton K., Yeatman H., Ma G. (2011). Improved iodine status in breastfeeding women following mandatory fortification. Aust. N. Z. J. Public Health.

[15] Jooste P., Upson N., Charlton K.E. (2005). Knowledge of iodine nutrition in the South African adult population. Public Health Nutr..

[16] Combet E., Bouga M., Pan B., Lean M.E.J., Christopher C.O. (2015). Iodine and pregnancy–a UK cross-sectional survey of dietary intake, knowledge and awareness. Br. J. Nutr..

[17] O’kane S.M., Pourshahidi L.K., Farren K.M., Mulhern M.S., Strain J.J., Yeates A.J. (2016). Iodine knowledge is positively associated with dietary iodine intake among women of childbearing age in the UK and Ireland. Br. J. Nutr..

[18] Charlton K.E., Gemming L., Yeatman H., Ma G. (2010). Suboptimal iodine status of Australian pregnant women reflects poor knowledge and practices related to iodine nutrition. Nutrition.

[19] Garnweidner-Holme L., Aakre I., Lilleengen A.M., Brantsæter A.L., Henjum S. (2017). Knowledge about iodine in pregnant and lactating women in the Oslo area, Norway. Nutrients.

[20] Liu S., Li M., Fan L., Liu P., Meng F., Su X., Zhang Z. (2019). Iodine Deficiency Disorders (IDD). Endemic Disease in China.

[21] Tai M., Tizhang L., Yubin T., Bingzhong C., Xianyi Z. (1982). The present status of endemic goitre and endemic cretinism in China. Food Nutr. Bull..

[22] Yao Y.-N., Yuan X.-L., Zhu J., Xiang L.-C., Li Q., Deng K., Li X.-H., Liu H.-M. (2021). Geographic variations in the incidence of congenital hypothyroidism in China: A retrospective study based on 92 million newborns screened in 2013-2018. Chin. Med. J..

[23] Shi X., Han C., Li C., Mao J., Wang W., Xie X., Li C., Xu B., Meng T., Du J. (2015). Optimal and safe upper limits of iodine intake for early pregnancy in iodine-sufficient regions: A cross-sectional study of 7190 pregnant women in China. J. Clin. Endocrinol. Metab..

[24] Han T., Dong J., Zhang J., Zhang C., Wang Y., Zhang Z., Xiang M. (2022). Nutrient supplementation among pregnant women in China: An observational study. Public Health Nutr..

[25] Vandenbroucke J.P., von Elm E., Altman D.G., Gøtzsche P.C., Mulrow C.D., Pocock S.J., Poole C., Schlesselman J.J., Egger M., STROBE Initiative (2014). Strengthening the Reporting of Observational Studies in Epidemiology (STROBE): Explanation and elaboration. Int. J. Surg..

[26] von Elm E., Altman D.G., Egger M., Pocock S.J., Gøtzsche P.C., Vandenbroucke J.P., STROBE Initiative (2014). The Strengthening the Reporting of Observational Studies in Epidemiology (STROBE) Statement: Guidelines for reporting observational studies. Int. J. Surg..

[27] Henjum S., Brantsæter A.L., Kurniasari A., Dahl L., Aadland E.K., Gjengedal E.L.F., Birkeland S., Aakre I. (2018). Suboptimal iodine status and low iodine knowledge in young Norwegian women. Nutrients.

[28] Yu Z., Zheng C., Zheng W., Wan Z., Bu Y., Zhang G., Ding S., Wang E., Zhai D., Ma Z.F. (2020). Mild-to-moderate iodine deficiency in a sample of pregnant women and salt iodine concentration from Zhejiang province, China. Environ. Geochem. Health.

[29] Yang Y., Wang G., Pan X. (2002). China Food Composition 2002.

[30] (2006). Method for Determination of Iodine in Urine by As3+-Ce4+ Catalytic Spectrophotometry. Health Standard of China..

[31] World Health Organization (WHO) (2007). Assessment of Iodine Deficiency Disorders and Monitoring Their Elimination: A Guide for Programme Managers.

[32] Liu S., Sharp A., Villanueva E., Ma Z.F. (2022). Breast milk iodine concentration (BMIC) as a biomarker of iodine status in lactating women and children < 2 years of age: A systematic review. Nutrients.

[33] Brough L., Jin Y., Shukri N.H., Wharemate Z.R., Weber J.L., Coad J. (2015). Iodine intake and status during pregnancy and lactation before and after government initiatives to improve iodine status, in Palmerston North, New Zealand: A pilot study. Matern. Child Nutr..

[34] Axford S., Charlton K., Yeatman H., Ma G. (2012). Poor knowledge and dietary practices related to iodine in breastfeeding mothers a year after introduction of mandatory fortification. Nutr. Diet..

[35] Bath S.C., Steer C.D., Golding J., Emmett P., Rayman M.P. (2013). Effect of inadequate iodine status in UK pregnant women on cognitive outcomes in their children: Results from the Avon Longitudinal Study of Parents and Children (ALSPAC). Lancet.

[36] Hynes K.L., Otahal P., Hay I., Burgess J.R. (2013). Mild iodine deficiency during pregnancy is associated with reduced educational outcomes in the offspring: 9-year follow-up of the gestational iodine cohort. J. Clin. Endocrinol..

[37] Xiao Y., Sun H., Li C., Li Y., Peng S., Fan C., Teng W., Shan Z. (2018). Effect of iodine nutrition on pregnancy outcomes in an iodine-sufficient area in China. Biol. Trace Elem. Res..

[38] Charlton K., Yeatman H., Lucas C., Axford S., Gemming L., Houweling F., Goodfellow A., Ma G. (2012). Poor knowledge and practices related to iodine nutrition during pregnancy and lactation in Australian women: Pre-and post-iodine fortification. Nutrients.

[39] Jin Y., Coad J., Zhou S.J., Skeaff S., Benn C., Brough L. (2021). Use of Iodine Supplements by Breastfeeding Mothers Is Associated with Better Maternal and Infant Iodine Status. Biol. Trace Elem. Res..

[40] Li M., Chapman S., Agho K., Eastman C.J. (2008). Can even minimal news coverage influence consumer health-related behaviour? A case study of iodized salt sales, Australia. Health Educ. Res..

[41] Wang Z., Wu Y., Shi Z., Song J., Wang G., Xu C., Song Q., Jin W., Cui X., Wu C. (2021). Association of iodine-related knowledge, attitudes and behaviours with urinary iodine excretion in pregnant women with mild iodine deficiency. J. Hum. Nutr. Diet..

[42] Tian W., Yan W., Liu Y., Zhou F., Wang H., Sun W. (2021). The status and knowledge of iodine among pregnant women in Shanghai. Biol. Trace Elem. Res..

[43] Teng W., Shan Z., Teng X., Guan H., Li Y., Teng D., Jin Y., Yu X., Fan C., Chong W. (2006). Effect of iodine intake on thyroid diseases in China. N. Engl. J. Med..

[44] Zhao H., Tian Y., Liu Z., Li X., Feng M., Huang T. (2014). Correlation between iodine intake and thyroid disorders: A cross-sectional study from the South of China. Biol. Trace Elem. Res..

[45] Gu F., Ding G., Lou X., Wang X., Mo Z., Zhu W., Zhou J., Mao G. (2016). Incidence of thyroid diseases in Zhejiang Province, China, after 15 years of salt iodization. J. Trace Elem. Med. Biol..

[46] Yan Y.R., Liu Y., Huang H., Lv Q.G., Gao X.L., Jiang J., Tong N.W. (2015). Iodine nutrition and thyroid diseases in Chengdu, China: An epidemiological study. QJM Int. J. Med..

[47] Yang L., Zheng R.S., Wang N., Zeng H.M., Yuan Y.N., Zhang S.W., Li H.C., Liu S., Chen W.Q., He J. (2017). Analysis of incidence and mortality of thyroid cancer in China, 2013. Zhonghua Zhong Liu Za Zhi.

[48] Dong W., Zhang H., Zhang P., Li X., He L., Wang Z., Liu Y. (2013). The changing incidence of thyroid carcinoma in Shenyang, China before and after universal salt iodization. Med. Sci. Monit. Int. Med. J. Exp. Clin. Res..

[49] Zou S., Wu F., Guo C., Song J., Huang C., Zhu Z., Yu H., Guo Y., Lu X., Ruan Y. (2012). Iodine nutrition and the prevalence of thyroid disease after salt iodization: A cross-sectional survey in Shanghai, a coastal area in China. PLoS ONE.

[50] Gupta P.M., Gahche J.J., Herrick K.A., Ershow A.G., Potischman N., Perrine C.G. (2018). Use of iodine-containing dietary supplements remains low among women of reproductive age in the United States: NHANES 2011–2014. Nutrients.

[51] Hess S., Zimmermann M., Torresani T., Bürgi H., Hurrell R. (2001). Monitoring the adequacy of salt iodization in Switzerland: A national study of school children and pregnant women. Eur. J. Clin. Nutr..

[52] Zimmermann M.B., Aeberli I., Torresani T., Burgi H. (2005). Increasing the iodine concentration in the Swiss iodized salt program markedly improved iodine status in pregnant women and children: A 5-y prospective national study. Am. J. Clin. Nutr..

[53] Andersson M., Aeberli I., Wüst N., Piacenza A.M., Bucher T., Henschen I., Haldimann M., Zimmermann M.B. (2010). The Swiss iodized salt program provides adequate iodine for school children and pregnant women, but weaning infants not receiving iodine-containing complementary foods as well as their mothers are iodine deficient. J. Clin. Endocrinol. Metab..

